# The ethics of advancing artificial intelligence in healthcare: analyzing ethical considerations for Japan’s innovative AI hospital system

**DOI:** 10.3389/fpubh.2023.1142062

**Published:** 2023-07-17

**Authors:** Amelia Katirai

**Affiliations:** Research Center on Ethical, Legal, and Social Issues, Osaka University, Suita, Osaka, Japan

**Keywords:** artificial intelligence, healthcare, ethics, Japan, AI Hospital, innovation

## Abstract

Public and private investments into developing digital health technologies—including artificial intelligence (AI)—are intensifying globally. Japan is a key case study given major governmental investments, in part through a Cross-Ministerial Strategic Innovation Promotion Program (SIP) for an “Innovative AI Hospital System.” Yet, there has been little critical examination of the SIP Research Plan, particularly from an ethics approach. This paper reports on an analysis of the Plan to identify the extent to which it addressed ethical considerations set out in the World Health Organization’s 2021 Guidance on the Ethics and Governance of Artificial Intelligence for Health. A coding framework was created based on the six ethical principles proposed in the Guidance and was used as the basis for a content analysis. 101 references to aspects of the framework were identified in the Plan, but attention to the ethical principles was found to be uneven, ranging from the strongest focus on the potential benefits of AI to healthcare professionals and patients (*n* = 44; Principle 2), to no consideration of the need for responsive or sustainable AI (*n* = 0; Principle 6). Ultimately, the findings show that the Plan reflects insufficient consideration of the ethical issues that arise from developing and implementing AI for healthcare purposes. This case study is used to argue that, given the ethical complexity of the use of digital health technologies, consideration of the full range of ethical concerns put forward by the WHO must urgently be made visible in future plans for AI in healthcare.

## 1. Introduction

Despite the ethical complexity of emerging digital health technologies such as artificial intelligence (AI), public and private investments in them are intensifying (1, 2). Developments in AI—“the science and engineering of creating intelligent machines that have the ability to achieve goals like humans via a constellation of technologies” (3)—have contributed to an unprecedented potential for massive amounts of health-related data to be processed. Applications of AI range from assistance in clinical decision-making to administrative support, and can aid in analyzing data ranging from medical images to personal health data retrieved from devices connected through the Internet of Things (4). These abilities create new incentives to agglomerate health data and for public-private partnerships to most efficiently extract value (5). Yet, recent research highlights major ethical issues in AI in healthcare, including concerns about privacy and data ownership, the risk of harm through biased systems and a lack of human oversight, and the need for provisions to support stakeholders if disruptions to healthcare occur, such as by providing training for healthcare professionals (HCPs) (6, 7).

The Japanese government is investing heavily in AI in healthcare through its shift towards “Society 5.0,” where AI is deployed to solve societal issues, providing support for an aging population and balancing the impact of a shrinking workforce (8, 9). Japan faces an urgent need to offset growing imbalances in its healthcare system as a result of a super-aging society, exacerbated through the Covid-19 pandemic (2). In 2020, the proportion of the population aged over 65 years was 28.6—a significantly higher percentage than in other highly industrialized societies such as in the United States (16.6 percent), France (20.8 percent), or Germany (21.7 percent), with neighboring South Korea at 16 percent. To this end, the Japanese government is working to create a regulatory environment favorable to developing AI and to public-private partnerships, and offers a useful case study yielding insights into the potential possibilities and pitfalls of such an approach (2).

A key component of Japan’s governmental investment is a Cross-Ministerial Strategic Innovation Promotion Program (SIP) for an “Innovative AI Hospital System” (8, 10–12). First outlined in 2018 with targets set for 2022, it includes a five-part plan for AI in healthcare. Elements of the plan include developing agglomerated medical databases; an AI-powered system to facilitate informed consent; using AI to support screening for diseases including cancer; creating exemplary “AI hospitals;” and encouraging collaborations between governmental, industry, and academic actors. The SIP promotes AI as beneficial to patients and to HCPs by increasing efficiency and reducing burden. Though it is one of the major structured programs for implementing AI in healthcare in Japan and represents a significant investment of public funds in AI, there has been little critical examination of its ethical dimensions.

AI increasingly crosses national borders as technological developments in one locale set precedents to be replicated in other countries. In the absence of “specific ethical principles for use of AI for health” globally, the World Health Organization [WHO; (13)] released their Guidance on the Ethics and Governance of Artificial Intelligence for Health in 2021, collating concerns and principles for the application of AI in healthcare elicited from and reviewed by external experts. In the Guidance, which additionally offers a framework for governance, the WHO proposes six ethical principles for AI in healthcare on autonomy, human well-being, transparency and explainability, responsibility and accountability, inclusiveness and equity, and responsive and sustainable systems.

Despite the urgency of the ethical issues posed by AI, both in Japan and outside of it, the implementation of ethical principles is largely left to the discretion of developers of AI technologies themselves, due to a lack of regulation (14). This means that an orientation to the ethics of AI from the point of conception of plans for its development is essential to ensure that AI is created and implemented in beneficial and not harmful ways. Yet, “medical AI applications have been found to sometimes be designed without any explicit ethical considerations” (14). Japan is an important case study through which to examine how ethical concerns are accounted for in the development of AI for healthcare, as it is a front-runner in its active promotion, and sets a key precedent on a global scale (2). Lessons from the Japanese context can be used to inform policy and practice in other countries seeking to advance AI for healthcare.

As Karimian et al. (15) have argued, “developers of AI algorithms must be vigilant regarding potential dangers.” These risks are heightened in the case of AI in healthcare, and it is essential that government documentation providing direction for the advancement of AI in healthcare reflect attunement to these risks. In light of this, given that the WHO Guidance sets an international standard for ethical AI in healthcare, and considering the importance of Japan’s SIP in its plans for AI in healthcare, this paper reports on an analysis of the most-recent SIP Research Plan at the time of this writing, to identify the extent to which the Plan reflects the ethical principles in the WHO Guidance. I argue that the Plan shows insufficient consideration of the ethics of AI in healthcare and contend that consideration of a broader range of ethical concerns must urgently be made visible in such plans for AI.

## 2. Methodology

A framework was constructed for a content analysis, based on the description of each of the ethical principles set out in the WHO Guidance on Ethics and Governance of Artificial Intelligence for Health. Subcodes were created for each principle based on their description in the Guidance. A total of 30 sub-codes were created (Table 1). This coding framework was then applied by the author to the original Japanese text of the SIP Research Plan on the “Innovative AI Hospital System” [AI(人工知能)ホスピタルによる高度診断・治療システム 研究計画] (10). While the first version of the Plan was released in 2018, the document has been regularly reviewed, with the April 25, 2022 analyzed here as it is the most recent version of the document at the point of analysis, and at this time of writing.^1^

**Table 1:** Coding framework created from the WHO Guidance.

Code	Item
**1**	Protecting human autonomy
1.1	Does not undermine human autonomy (humans should remain in control)
1.2	Ensure that providers have the information necessary to make safe, effective use of AI systems
1.3	People understand the role that such systems play in their care
1.4	Protection of privacy and confidentiality
1.5	Valid informed consent obtained through appropriate legal frameworks for data protection
**2**	Promoting human well-being and safety and the public interest
2.1	Should not harm people
2.2	Should satisfy regulatory requirements for safety, accuracy and efficacy for well-defined use cases or indications
2.3	Measures of quality control in practice and quality improvement are available
2.4	Should not result in mental or physical harm that could be avoided by use of an alternative practice or approach
**3**	Ensuring transparency, explainability, and intelligibility
3.1	Should be intelligible or understandable to developers, medical professionals, patients, users, and regulators
3.2	Transparency – sufficient information published or documented before the design or deployment of an AI technology
3.3	Transparency – information facilitates meaningful public consultation and debate on how the technology is designed and how it should or should not be used
3.4	Explainable – explained according to the capacity of those to whom they are explained
**4**	Fostering responsibility and accountability
4.1	Clear, transparent specification of the tasks that systems can perform – stakeholders ensure that they can perform those tasks and that AI is used under appropriate conditions
4.2	Human warranty – evaluation by patients and clinicians in the development and deployment of AI
4.3	Regulatory principles applied upstream and downstream of the algorithm through human supervision
4.4	Accountability – appropriate mechanisms for questioning and redress for individuals and groups that are adversely affected by decisions based on algorithms
**5**	Ensuring inclusiveness and equity
5.1	Designed to encourage the widest possible appropriate, equitable use and access, irrespective of age, sex, gender, income, race, ethnicity, sexual orientation, ability or other characteristics protected under human right codes
5.2	Should be shared as widely as possible
5.3	Should be available for use not only in contexts and needs in high-income settings but also in the contexts and for the capacity and diversity of LMIC
5.4	Should not encode biases to the disadvantage of identifiable groups, especially groups that are already marginalized
5.5	Minimize inevitable disparities in power that arise between providers + patients, between policy-makers and people, between companies and governments
5.6	Monitored and evaluated to identify disproportionate effects on specific groups of people
5.7	Should not sustain or worsen existing forms of bias and discrimination
**6**	Promoting AI that is responsive and sustainable
6.1	Continuously, systematically, and transparently assess AI applications during actual use
6.2	Determine whether AI responds adequately and appropriately and according to communicated, legitimate expectations and requirements
6.3	Consistent with wider promotion of the sustainability of health systems, environments, and workplaces
6.4	Designed to minimize environmental consequences and increase energy efficiency
6.5	Consistent with global efforts to reduce the impact of human beings on the Earth’s environment, ecosystems, and climate
6.6	Governments and companies to address anticipated disruptions in the workplace, including training for health-care workers to adapt to the use of AI systems and potential job losses

A modified version of directed content analysis as proposed by Hsieh and Shannon (16) was used, through which the number of sentences within the Plan which reflected an orientation towards the ethical principles included in the framework above (Table 1) was tabulated. Where there were multiple phrases with a common code in a single sentence, these were collectively coded as one instance. Due to the structure of the original principles, some of the subcodes included in different principles overlapped, and where a sentence could potentially be coded under multiple subcodes, it was coded under a single subcode which, through reference to the original guidelines, appeared to best fit the broader principle. Where a particular sentence matched a broader principle but not a specific subcode, it was coded as a part of the broader principle. These results were then collated to indicate how frequently each component of the principles was referenced in the guidelines. The results are reported in Table 2, wherein “frequency” refers to the number of references in the Plan to a particular component of each of the WHO principles, as operationalized for this study. “Total by principle” refers to the number of total references to all components of a particular principle, to allow for comparison in the frequency of reference to each principle. It is noteworthy that neither the WHO Guidance nor its principles were directly referred to at any point in the Plan. Instead, all references tabulated here were indirect references to the principles. The results of this analysis are reported below, with all translations by the author.

**Table 2:** Frequency of references to principles in the WHO Guidance.

Code	Item	Frequency	Total by principle
**1.0**	Protecting human autonomy	0	14
1.1	Does not undermine human autonomy (humans should remain in control)	0	
1.2	Ensure that providers have the information necessary to make safe, effective use of AI systems	0	
1.3	People understand the role that such systems play in their care	0	
1.4	Protection of privacy and confidentiality	14	
1.5	Valid informed consent obtained through appropriate legal frameworks for data protection	0	
**2.0**	Promoting human well-being and safety and the public interest	43	44
2.1	Should not harm people	0	
2.2	Should satisfy regulatory requirements for safety, accuracy and efficacy for well-defined use cases or indications	1	
2.3	Measures of quality control in practice and quality improvement are available	0	
2.4	Should not result in mental or physical harm that could be avoided by use of an alternative practice or approach	0	
**3.0**	Ensuring transparency, explainability, and intelligibility	0	12
3.1	Should be intelligible or understandable to developers, medical professionals, patients, users, and regulators	0	
3.2	Transparency – sufficient information published or documented before the design or deployment of an AI technology	3	
3.3	Transparency – information facilitates meaningful public consultation and debate on how the technology is designed and how it should or should not be used	7	
3.4	Explainable – explained according to the capacity of those to whom they are explained	2	
**4.0**	Fostering responsibility and accountability	0	13
4.1	Clear, transparent specification of the tasks that systems can perform – stakeholders ensure that they can perform those tasks and that AI is used under appropriate conditions	0	
4.2	Human warranty – evaluation by patients and clinicians in the development and deployment of AI	13	
4.3	Regulatory principles applied upstream and downstream of the algorithm through human supervision	0	
4.4	Accountability - appropriate mechanisms for questioning and redress for individuals and groups that are adversely affected by decisions based on algorithms	0	
**5.0**	Ensuring inclusiveness and equity	5	18
5.1	Designed to encourage the widest possible appropriate, equitable use and access, irrespective of age, sex, gender, income, race, ethnicity, sexual orientation, ability or other characteristics protected under human right codes	0	
5.2	Should be shared as widely as possible	12	
5.3	Should be available for use not only in contexts and needs in high-income settings but also in the contexts and for the capacity and diversity of LMIC	0	
5.4	Should not encode biases to the disadvantage of identifiable groups, especially groups that are already marginalized	0	
5.5	Minimize inevitable disparities in power that arise between providers + patients, between policy-makers and people, between companies and governments	0	
5.6	Monitored and evaluated to identify disproportionate effects on specific groups of people	0	
5.7	Should not sustain or worsen existing forms of bias and discrimination	1	
**6.0**	Promoting AI that is responsive and sustainable	0	0
6.1	Continuously, systematically, and transparently assess AI applications during actual use	0	
6.2	Determine whether AI responds adequately and appropriately and according to communicated, legitimate expectations and requirements	0	
6.3	Consistent with wider promotion of the sustainability of health systems, environments, and workplaces	0	
6.4	Designed to minimize environmental consequences and increase energy efficiency	0	
6.5	Consistent with global efforts to reduce the impact of human beings on the Earth’s environment, ecosystems, and climate	0	
6.6	Governments and companies to address anticipated disruptions in the workplace, including training for health-care workers to adapt to the use of AI systems and potential job losses	0	
	Total	101	101

## 3. Results

In total, there were 101 references to aspects of the WHO principles in the SIP Plan, but attention to the principles was notably uneven. The number of references to each aspect of the principles is reported in Table 2. Each principle will be examined in turn below, in order of frequency.

Overall, there was the most attention (*n* = 44) to Principle 2, “Promoting human well-being and safety and the public interest,” through statements focused on the expectations that AI could benefit stakeholders. For example, among the 43 coded items, there were 16 references (pp. 1, 2, 3, 7, 10, 13, 22, 28, 33, 34, 37, 42, 49) to the expectation that AI would reduce burden—primarily the burden experienced by HCPs, but also that of patients—4 references to increased efficiency (pp.10, 22, 26, 42), and 4 references to the benefits of AI in healthcare in a super-aging society (pp. 1, 10, 11, 42). Moreover, there were notable references to AI as a resource in times of disaster (p. 8), and to the socio-economic benefits of improved patient health and its knock-on effects on the labor force (p. 11). A representative example is Extract 1 below:

*In addition, these technologies will be used to reduce the burden on healthcare professionals, including doctors and nurses, in hospitals, and to increase the efficiency of medical expenses, thereby contributing to overcoming various issues in a super-aging society, and to economic development.* Extract 1 (p. 1)

This situated AI within the broader context of the problems faced by the Japanese health system and positioned it as a potential solution to these issues. However, the focus on efficiency and burden reflected a narrow representation of the issues in the healthcare system. Moreover, its subcodes (see Table 1), including the risk of direct or indirect harm—particularly forms of harm that could be avoided by using alternatives to AI—were insufficiently addressed. There was also a lack of attention to regulatory requirements or measures of quality control.

Principle 5 (“Ensuring inclusiveness and equity;” *n* = 18) was the next most frequent, though here again, coverage of the items was uneven. 4 instances were coded under Principle 5 more broadly (pp. 16, 19, 21, 25), as they primarily addressed ensuring linguistic inclusivity through Natural Language Processing systems. Also coded under Principle 5 were calls to expand the reach of the AI systems by making them available for use outside of Japan, with 5 references to this (pp. 6, 7, 19, 35, 50). However, it is unclear whether the motivations for this were based on ethical ideals, or due to the potential commercial benefits of such initiatives, as in Extract 2 below:

*At the end of the project, this model will be used as a basis for industrialization through overseas expansion, etc.* Extract 2 (p. 35)

Though the need to avoid creating inequality of access and of quality was acknowledged, sharing technologies with resource-poor locales globally, such as with low-and middle-income countries, went unaddressed. Moreover, there was little consideration of the potential for bias and discrimination, apart from two references to using AI to prevent inequity in the quality of healthcare (pp.3, 28).

There were 14 references to Principle 1, “Protecting human autonomy,” the references to which focused solely on the “protection of privacy and confidentiality.” Within this, in turn, privacy and confidentiality were narrowly dealt with, focusing primarily on ensuring secure systems. This does not sufficiently reflect how privacy and confidentiality are conceptualized as duties which are a part of respect for autonomy, and instead reflects a narrow approach to both autonomy, and to privacy itself, given that there was little consideration of other aspects of autonomy such as patient centeredness or control in decision-making (15). The Plan referred to the European General Data Protection Regulation and to potential differences between Japan and other contexts where the systems may eventually be applied, but without framing from the perspective of autonomy (Extract 3).

*When international expansion is in view, the handling of the sensitive information of international persons will be considered according to international standards; it is important that our country retain control of collaboratively developed platforms without being overly concerned with competitiveness.* Extract 3 (p. 4)

A notable absence in this area was around ensuring that appropriate consent is gained for the use of patient data. For example, diagrams (pp. 17, 18) which depict the flow of patient data into databases and their retrieval for use did not depict patient consent being obtained. Interestingly, though one aim in the SIP was to use AI to help facilitate informed consent for medical procedures, there was little attention to consent for data used for the systems themselves.

Similarly, though there were 13 references to Principle 4, these were concentrated in one area: “providing human warranty through evaluation by patients and clinicians in the development and deployment of AI.” This included ensuring evaluation of the systems developed through the Plan both prior to their development, at the end of each fiscal year, and a final evaluation at an unspecified time, which would include evaluation of necessity, efficiency, and efficacy (p.48). It is noteworthy as well that one component of this was the establishment of a board to consider the Ethical, Legal, and Social Issues (ELSI) of the technologies (pp. 5, 15, 44, 48; Extract 4). However, specifics about the board were not provided in the Plan, and online searches have not yet yielded easily accessible details at this time.

*In addition to self-evaluation and PD and sub-PD evaluations, an oversight committee, an evaluation committee made up of third-party members, an intellectual property oversight committee, and a committee for evaluation of research and development from the perspective of Ethical, Legal, and Social Issues (hereinafter referred to as the ELSI Committee) as well as a Project Management Office (PJMO) will be established to evaluate and manage the PDCA cycle internally and externally.* Extract 4 (p. 5)

It was unspecified how accountability and responsibility for the systems would be handled, and if provisions would be made in advance for this.

There were 12 instances in which Principle 3 were addressed. Compared to the other categories, these were more evenly distributed among the subcomponents. Commitments were made to share information about the development of the technologies covered by the project, (e.g., pp. 41, 48), as well as a commitment to ensure patient understanding of the technologies (e.g., pp. 28, 32). However, this was not directly linked to the public more broadly (Extract 5).

*In addition, by appropriately including the opinions of patients and users, and establishing an organization to consider system design optimized to society and regulations that pose obstacles, hearings and negotiations will be conducted with relevant government ministries and agencies.* Extract 5 (p. 45)

It is also noteworthy here that regulation was described in the extract above as a potential obstacle.

There were no instances reflecting Principle 6, “Promoting AI that is responsive and sustainable.” It is notable that some aspects of the principle—namely, evaluation of “whether AI responds adequately and appropriately and according to communicated, legitimate expectations and requirements”—overlap with other aspects of the principles, such as providing for human warranty and evaluation by patients and clinicians (Principle 4) and meeting regulatory requirements (Principle 2). However, there were no references to these points in the Plan from the perspective of sustainability or responsiveness. And finally, there was no attention given to: the environmental impact of the technologies; considering possible impact of new technologies on employment; considering potential disruptions in healthcare workflows; or educational or other provisions to equip HCPs to handle these changes.

## 4. Discussion

Close attention to the ethics of AI in healthcare is imperative, as evidenced by the creation of the WHO Guidance itself (13). The results of this study have brought to light an uneven approach to ethics in the SIP Innovative AI Hospital System Research Plan, and a narrow conception within the Plan of the potential ethical issues of the technologies it proposes. The strongest focus in the Plan is placed on how the proposed technologies can promote “human well-being and safety and the public interest” (Principle 2). Yet, this is narrowly defined and primarily concentrated on reducing burden on HCPs, and on increasing efficiency. Given Japan’s “super-aging society” (2), these are undoubtedly key goals for the medical system, but this emphasis on efficiency may impose further pressure on already overworked healthcare professionals HCPs.

Moreover, the Plan reflects a narrow and optimistic focus on the positive impact of the technologies, with little delineation of how this will be reached. For example, the Plan did not specify how the introduction of the technologies will directly link to reduced burden for HCPs, and how reduced burden will in turn bring benefits to HCPs and their patients. It also disregards the new skills that HCPs may need in order to effectively work with AI and side-steps the question of from where these skills will be obtained and how, and the potential for this to create additional burden.

Furthermore, it is unclear from the Plan how the proposed technologies were selected for such focused implementation, and whether the areas of development are indeed top priorities for Japan’s medical system. Topol (3) and Keane and Topol (17) problematize the promotion of technologies for healthcare without ensuring that they bring clinical benefit and improvements to the status quo. There is a particular need for close examination considering the prevalence of “vaporware” —technologies which do not exist and/or do not perform as intended—among proposed uses of AI (18). The positive approach in the Plan further suggests a technological solutionist approach to the problems of healthcare, which expects that the introduction of new technologies can resolve fundamental issues, particularly in relation to overburdened healthcare workers and a lack of sufficient resources (1). Rather, the claims made for AI in healthcare should be critically examined, alongside consideration of what other societal shifts may be needed to support healthcare workers, beyond the introduction of new technologies (19).

In addition, there was little consideration of the potential direct or indirect harm which could occur as a result of the use of AI, as called for under Principle 2. There is a need for the consideration of proportionality, through which the application of new technology should be commensurate with its potential risks, particularly in relation to long-term social, economic, and environmental sustainability (20).

Bias in AI systems may lead to significant harm and discriminatory outcomes (Principle 5). This was not addressed sufficiently in the Plan. There was no description of attempts to ensure the reduction of bias or to avoid discriminatory outcomes. This is problematic in light of the discriminatory impact of AI in healthcare, which can affect patient well-being and mortality (21, 22). In the United States, for example, the use of AI has resulted in the allocation of resources along racial lines, disadvantaging already vulnerable populations (21). These oversights are particularly worrying in Japan, given that it “has the lowest percentage of foreign-born residents in the world among developed nations, suggesting far fewer cases and thus less experience working with non-nationals” (2). Thus, algorithms developed in and based on data from the Japanese context can be expected to lack sufficient diversity, and risk perpetuating healthcare inequality for minorities. Moreover, though the Plan includes provisions for securing access to patient data, the development of the systems it calls for appears to be moving forward without consideration of the need for such data to be representative. Thus, explicit provisions to avoid bias and discriminatory outcomes are necessary but lacking in the Plan. This is especially important if technologies are exported to other contexts, as described in the Plan itself. Here, it is important to note that one of the goals of AI implementation described in the Plan is to make healthcare more accessible to non-Japanese speakers, by reducing potentially fatal language barriers (23). If expanded further, this could bring benefits to immigrants and non-Japanese populations, particularly given that immigrants continue to face barriers to access for “ambulatory and emergency care,” even with insurance coverage (23).

Autonomy (Principle 1) is another area where the Plan takes a narrow focus, as the preservation of human autonomy as stipulated in the WHO principles was not addressed in the Plan beyond limited consideration of data security and privacy, with a notable lack of consideration about the need for appropriate consent. Although, as stipulated by the WHO, “[r]espect for autonomy also entails the related duties to protect privacy and confidentiality and to ensure informed, valid consent by adopting appropriate legal frameworks for data protection,” the Plan focuses primarily on data security, without consideration of the need to preserve patient autonomy. By situating privacy concerns under Principle 1, “Protecting human autonomy,” the WHO Guidance points to how privacy and confidentiality ensure human autonomy on both sides of clinical interactions, which is critical to well-functioning healthcare systems (19). However, the protection of privacy is not synonymous with autonomy, which can be understood as the ability to act “in accordance with one’s goals and values” (24) It is noteworthy that research in other settings has also found that narrower issues of data security and privacy are often more frequently addressed in considerations of AI ethics than principles such as autonomy (25). In this case, the absence of direct attention to autonomy in the Plan may be grounded in an expectation in the Japanese context that AI-based systems remain supplemental to human HCPs, providing for the “override” on decisions as called for in the WHO Guidance (2), but does not necessarily ensure patient autonomy. Moreover, in light of recent work by Kodera et al. (26) which suggests that this HCP-centric approach may change, it is essential that stipulations to preserve autonomy be clearly put forward.

Care is needed in the handling of patient data, given the risks presented by rising numbers of cyber-attacks against healthcare facilities (27). Davis (27) highlights the risk of “function creep,” through which data collected for one purpose comes to be used for another. Data breaches can reveal sensitive healthcare data, which may be used against data subjects in consequential settings including employment and for insurance judgments (28). This highlights the need for consideration of privacy issues. Patient consent for the use of data is also relevant here. Facilitating informed consent is presented in the Plan as an end goal for the development of AI, rather than to ensure that the data used for AI itself is ethically obtained.

Accountability is additionally a major issue in relation to healthcare, particularly given the black-boxed nature of many algorithms (3). How accountability would be handled and who would be responsible for potential issues that arose—such as when problematic decisions were influenced or made by AI—were insufficiently addressed. Research further suggests that these are important considerations for patients in relation to their willingness to engage with AI in healthcare (29, 30).

There were provisions in the Plan for consultation with direct stakeholders, including patients, which reflects trends towards the democratization of healthcare, and recognition of the value of patient involvement in healthcare (31–33). However, this did not extend to the level of broader “public consultation and debate” on the technologies, called for in the WHO Guidance. Caution is needed in this area, as there is the risk that consultation with limited stakeholder groups can lead to a form of “participation-washing” (34), in which the perspectives of small numbers of participants are overgeneralized to represent public perspectives. In this area as well, provisions for including the perspectives of minority users of healthcare would be desirable.

And finally, there was a significant lack of consideration of Principle 6, and especially for ensuring the sustainability—broadly defined—of healthcare. As discussed above, social sustainability was insufficiently addressed, and the Plan lacked provisions to offset potential disruptions in healthcare, such as through adequate training or education. Moreover, the environmental consequences of AI implementation raised in Principle 6 were not considered. Van Wynsberghe (35) describes the development of AI ethics as occurring in three waves. In this model, the current second wave of AI ethics is concerned with the potential for the amplification of existing biases in healthcare, while the coming, third-wave of AI ethics is concerned with the sustainability of AI systems. As Van Wynsberghe (35), Crawford (36), Brevini (37), and Jaume-Palasi (38) have argued, AI creates a substantial environmental burden across its life course, ranging from extracting materials such as rare earth metals and lithium used in the hardware, to the carbon emissions in creating and using systems and their data centers. Given the urgency of the breach of planetary boundaries and the extreme degradation of the global environment, all projects, including this one, must include consideration of their environmental impact.

### 4.1. Limitations and future directions

Ultimately, this exploratory analysis highlights significant oversights and a need for greater awareness of the potential ethical issues around AI in healthcare in the SIP, which are insufficiently considered despite the scale and governmental backing of the SIP. Although the Plan did provide for the outsourcing of ethical consideration through the creation of an ELSI Committee, lack of attention to the risks of bias and discrimination, of privacy and consent, and of the sustainability of the proposed technologies were problematic oversights in the context of broader debates on the ethics of AI. Plans for the development of AI in healthcare must contain explicit consideration of and provisions to offset a range of ethical issues. Though this study focused on a Japanese case, it highlights a need for similar, critical examination of plans set in other contexts. Japan’s status as a front-runner for the implementation of AI into healthcare allows it to serve as an exemplar, enabling other countries to avoid possible pitfalls through lessons learned from the Japanese case.

This study was an exploratory analysis of ethical considerations in the Plan. It is noteworthy that the frequency of reference to a particular principle is just one possible metric and does not necessarily imply that a principle is perceived to be important or unimportant. Moreover, given the complexity of ethical principles and their application, reference to a principle in the Plan does not necessarily reflect the extent to which it is acted on in practice. Furthermore, is possible that further ethical consideration may have been conducted under the purview of the ELSI Committee described above, or within the design and implementation of the individual technologies called for in the Plan. Indeed, given that the Plan promotes the necessity of the technologies, the omission of direct attention to ethical considerations may strengthen the perceived merit of the technologies. Yet, consideration of the ethics of emerging technologies is essential in ensuring the longer-term social acceptance, trustworthiness, and beneficence of the proposed technologies. Particularly as the period allotted for the SIP draws to a close, further research may build on this exploratory study to examine the extent to which ethical issues were considered in the actual execution of the Plan and explore whether plans for AI in healthcare developed in other contexts share similar oversights.

## Data availability statement

The raw data supporting the conclusions of this article will be made available by the authors, without undue reservation.

## Author contributions

The author confirms being the sole contributor of this work and has approved it for publication.

## Funding

This work was funded by the Japan Science and Technology Agency Research Institute of Science and Technology for Society Grant Number JPMJRX19H1.

## Conflict of interest

The author declares that the research was conducted in the absence of any commercial or financial relationships that could be construed as a potential conflict of interest.

## Publisher’s note

All claims expressed in this article are solely those of the authors and do not necessarily represent those of their affiliated organizations, or those of the publisher, the editors and the reviewers. Any product that may be evaluated in this article, or claim that may be made by its manufacturer, is not guaranteed or endorsed by the publisher.

## Footnotes

^1^https://www8.cao.go.jp/cstp/gaiyo/sip/keikaku2/10_aihospital_1.pdf
